# Supplementary figures and images for: A Combination of Glutaminase Inhibitor 968 and PD-L1 Blockade Boosts the Immune Response against Ovarian Cancer
Source: Biomolecules. 2021 Nov 23;11(12):1749. doi: 10.3390/biom11121749 (PMC8698585; doi:10.3390/biom11121749)

**Supplementary Figure S1.** Correlation between GLS and immunosuppressive genes.

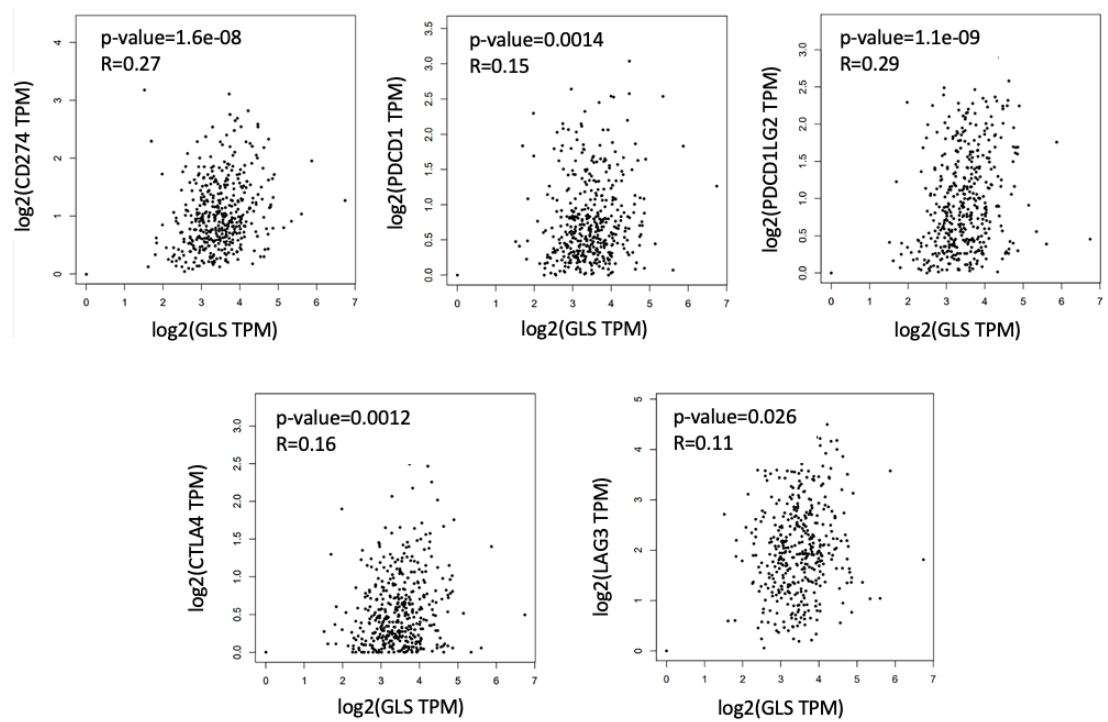

Supplement: Supplementary file 1 [file biomolecules-11-01749-s001.zip › biomolecules-1351073-supplementary.pdf]
